# Revealing proteins associated with symbiotic germination of *Gastrodia elata* by proteomic analysis

**DOI:** 10.1186/s40529-018-0224-z

**Published:** 2018-03-06

**Authors:** Xu Zeng, Yuanyuan Li, Hong Ling, Juan Chen, Shunxing Guo

**Affiliations:** Institute of Medicinal Plant Development, Chinese Academy of Medical Sciences, Beijing, 100193 People’s Republic of China

**Keywords:** *Gastrodia elata*, Mycorrhizal fungi, Symbiotic germination, Plant defense

## Abstract

**Background:**

*Gastrodia elata*, a mycoheterotrophic orchid, is a well-known medicinal herb. In nature, the seed germination of *G. elata* requires proper fungal association, because of the absence of endosperm. To germinate successfully, *G. elata* obtains nutrition from mycorrhizal fungi such as *Mycena*. However, *Mycena* is not able to supply nutrition for the further development and enlargement of protocorms into tubers, flowering and fruit setting of *G. elata*. To date, current genomic studies on this topic are limited. Here we used the proteomic approach to explore changes in *G. elata* at different stages of symbiotic germination.

**Results:**

Using mass spectrometry, 3787 unique proteins were identified, of which 599 were classified as differentially accumulated proteins. Most of these differentially accumulated proteins were putatively involved in energy metabolism, plant defense, molecular signaling, and secondary metabolism. Among them, the defense genes (e.g., pathogenesis-/wound-related proteins, peroxidases, and serine/threonine-protein kinase) were highly expressed in late-stage protocorms, suggesting that fungal colonization triggered the significant defense responses of *G. elata*.

**Conclusions:**

The present study indicated the metabolic change and defensive reaction could disrupt the balance between *Mycena* and *G. elata* during mycorrhizal symbiotic germination.

## Background

In nature, orchid seeds possess no endosperm therefore are devoid of nutrient supply. Mycorrhizal fungi provide the orchid seeds with signals and nutrients for germination, a mechanism named symbiotic germination. After germination, the orchid seeds give rise to protocorms. The protocorm is a post-embryonic structure from which both shoot and root systems subsequently differentiate. After the differentiation of green leaves, most orchid seedlings acquire autotrophy, while some orchids are achlorophyllous and obtain their entire carbon source from their mycorrhizal fungi. These orchids are known as fully mycoheterotrophic plants (Leake 2004; Dearnaley 2007).

*Gastrodia elata*, a fully mycoheterotrophic orchid, associates with two groups of fungal partners, *Mycena* and *Armillaria*. The ontogenesis of *G. elata* has four stages: seed germination, tuber formation, flowering and fruiting. *Mycena* species acts as its first symbiont during the early-stage of seed germination and protocorm development (Kim et al. 2006). However, *Mycena* cannot provide a regular supply of nutrients for further development of *G. elata* during the late-stage of protocorm development. Once a protocorm has been formed, *G. elata* switches its association to the second symbiont *Arminaria*, which subsequently invades the adult rhizome rapidly. For the enlargement of tubers, flowering, and fruit setting, the *Armillaria* becomes essential for the nutrient supply (Tsai et al. 2016). For more than 30 years, our research group only identified some *Mycena* species (e.g. *M. dendrobii*, *M. orchidicola*, *M. anoectochila*, and *M. osmundicola*) which were able to promote the germination of *G. elata*. In addition, *G. elata* is a well-known Chinese medicinal plant. Recent studies have indicated that it had strong potential to combat Alzheimer’s disease, Parkinson’s disease, and other neurodegenerative diseases (Manavalan et al. 2012).

The researches of the plant-fungus interactions in orchid mycorrhiza are still limited (Dearnaley 2007). Most studies agreed that the orchid mycorrhiza deserved more attention because arbuscular mycorrhiza (Hogekamp et al. 2011) and ectomycorrhiza (Plett and Martin 2011) had been profoundly studied. Recently, Perotto et al. (2014) investigated gene expression in mycorrhizal orchid protocorms (*Serapias vomeracea* colonized by *Tulasnella calospora*) to understand the molecular bases of plant-fungus interactions. Furthermore, Valadares et al. (2014) performed 2D-LC–MS/MS coupled to isobaric tagging for relative and absolute quantification and identify proteins with differential accumulation in *Oncidium sphacelatum* at different stages of mycorrhizal protocorm development. These studies suggested that the protocorm was a relatively constant period in all stage of orchid ontogenesis. It could represent a more feasible experimental system to analyze molecular and cellular aspects of the early plant-fungus interactions of orchid mycorrhiza. By contrast, orchids at maturity have a complicated symbiotic relationship, depending largely upon the trophic strategy of the host plant species and on the environmental conditions.

In this study, we investigated the changes of proteomic profiles during the symbiotic seed germination of *G. elata* inoculated with *M. dendrobii*. Our results may be useful for elucidating the reasons for partnership change during *G. elata* seed germination.

## Methods

### Plant materials

**Fig. 1:**
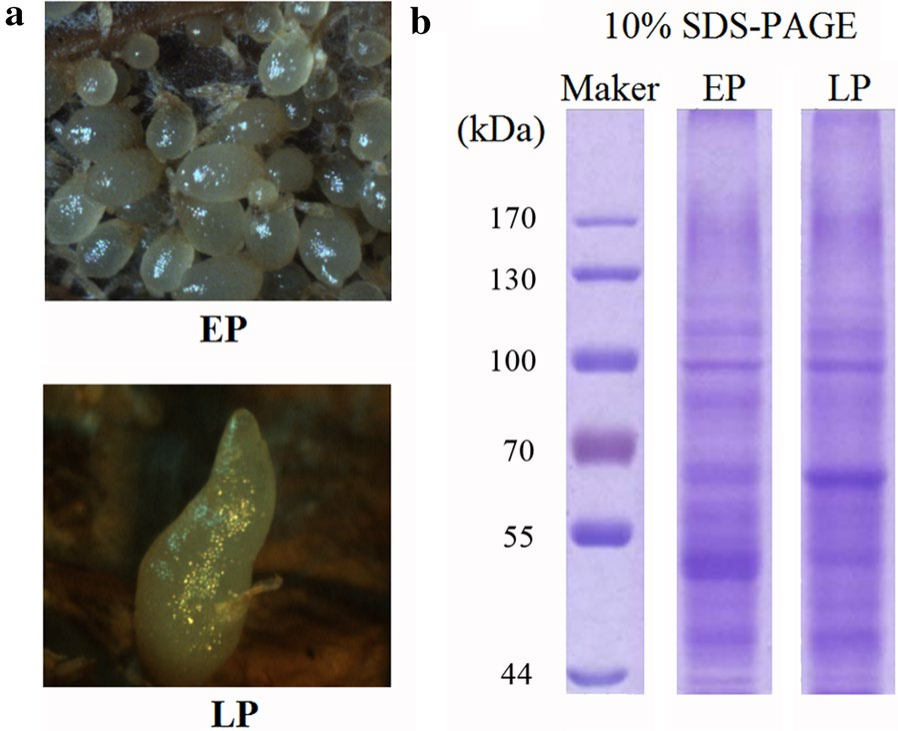
**a** The early-stage and the late-stage protocorms (EP and LP) of *G. elata*; **b** 10% SDS-PAGE of total protein

The flowers of *G. elata* were pollinated by hand in the production base at Shaanxi, China. After 16 days of pollination, capsules were collected just prior to dehiscence. The mycorrhizal fungal isolate (*M. dendrobii*) was incubated on fresh Potato Dextrose Agar (PDA: potato infusion, boiling 200 g potato in 1000 ml water; 20 g dextrose; 15 g agar powder) in the dark at 25 °C. The procedure for symbiotic germination of *G. elata* was performed according to the description in a previous report (Kim et al. 2006). Briefly, the culture media for *M. dendrobii* was prepared by fallen leaves of *Quercus* and rice bran at the ratio of 8:2 (V/V). Culturing in dark was benefit to the growth of *M. dendrobii* for 4 weeks at 25 °C. After 4 weeks of incubation, the culture media were fully colonized by *M. dendrobii*. Leaves infested with *M. dendrobii* were placed on fresh PDA and spread on several hundred seeds of *G. elata*, and then dark-cultured for 4–8 weeks at 25 °C. Germination performance was evaluated weekly under a stereo-microscope for 2 months. The early-stage of protocorms (EP) and the late-stage of protocorms (LP) were carefully collected under a stereo-microscope (Fig. 1). For further proteomic study, samples of equal fresh weight of EP and LP of *G. elata* were collected and immediately stored at − 80 °C.

### Protein extraction, digestion, iTRAQ labeling

Protein extraction, digestion, iTRAQ labeling, and mass spectrometry were conducted using protocols from previous paper (Xing et al. 2014). Briefly, total protein was extracted from each sample using Plant Total Protein Extraction Kit PE0230 (Sigma-Aldrich, USA) according to the manufacturer’s instructions. Bradford method was selected to determine the protein content. Proteins of each sample were detected by 10% SDS-PAGE (Fig. 1). Each of the samples (75 μg in total) was deoxidized with 20 mM DTT, alkylated with 50 mM IAA and digested with trypsin.

The peptide samples were labeled using the iTRAQ 4-plex kit (AB sciex, USA) according to the manufacturer’s protocol. Then, EP and LP were labeled by 116 and 117 iTRAQ, respectively. After labeling, the equal amounts of each sample were mixed together and lyophilized.

### Mass spectrometry

The pooled mixture from labeled samples was dissolved in mobile phases A and fractioned by Durashell RP column (5 µm, 150 Å, 250 mm × 4.6 mm, Agela) from L-3000 HPLC system (Rigol, China). Eluent was collected every minute, pooled into 12 samples and dried under vacuum. Peptides were eluted from the C18 analytical column with a 40 min gradient at a speed of 350 nl/min on an Eksigent Ultra HPLC (AB sciex, USA). The mass spectrum conditions for Triple TOF 5600 was set as follow: The spray voltage was set at 2.5 kV and the temperature of heater was 150 °C. The mass spectrum scan range was *m/z* 350–1250 and the tandem mass spectrometry (MS/MS) scan range was *m/z* 100–1500.

### Database search and functional annotation

Raw proteome data were searched using the Proteinpilot™ v4.0 search engine with percolator against the *G. elata* unigene translation database, including 9908 sequences. Unigene translation sequences were obtained from *G. elata* protocorm transcriptome sequences (Zeng et al. 2017). Based on RNA-seq, the clean reads from protocorm libraries were pooled together and *denovo* assembled into 139,756 unigenes, including 42,140 well-annotated unigenes. Finally, 9908 unigenes were able to translate into protein sequences and establish the self-built database (unigene translation database). The average iTRAQ ratios and standard deviations were calculated for each protein using all of the available treatment control iTRAQ pairs. A 1.5-fold cutoff was used to determine up-accumulated or down-accumulated proteins, with a *P* value of < 0.05.

Functional annotation of the proteins was performed using GO and KEGG annotation. Gene Ontology (GO) analyses were performed by WEGO (Web Gene Ontology Annotation Plot, http://wego.genomics.org.cn/) for plotting GO annotation results (Ye et al. 2006; Zeng et al. 2017). KEGG is a database for recoding the collection of high-level functions and the utility of the biological system. Here, KOBAS software was used for the statistical of DAPs in KEGG pathways (Kanehisa et al. 2008).

### Quantitative PCR

Total RNA of seed and protocorm samples was extracted using RNeasy^®^ Plant Mini Kit (QIAGEN, Germany) according to the manufacturer’s instructions. Primers designed with Primer Premier 5.0 are shown in Table 1. A PrimerScoript™ RT reagent Kit (TaKaRa, Japan) was used for reverse transcription. First, 1 μl RT product diluted with 20 μl ddH_2_O was used as a template. Then, qPCR was performed in 15 µl reaction mixture containing 7.5 µl of 2× SYBR^®^ Premix Ex TaqTM II (TaKaRa, Japan), 1.5 µl of cDNA template, and 0.3 µl of each gene specific primers. Overall, we preformed three biological replicates and three technical replicates using the LightCycler^®^ 480 II RT-PCR System (Roche, Switzerland). The parameters for the reactions were: 95 °C for 30 s, 40 cycles of 95 °C for 5 s, and 60 °C for 30 s. The cDNA libraries were standardized to housekeeping gene 18S rRNA. The 2^−ΔΔCt^ method was used for evaluating gene expression.

**Table 1:** The quantitative PCR primers of putative genes

Gene ID	Forward primer (5′–3′)	Reverse primer (5′–3′)
18S rRNA	CCAGGTCCAGACATAGTAAG	GTACAAAGGGCAGGGACGTA
c48836	AACCTCTTCCGCCATACCTGC	GCTCTCCGCTTCAACTGACCA
c54199	GGCGTTGTGGAGAGCATTGGA	TTTGTCCGTGCCATGCCTTTCA
c81881	ATGCCGCCTCGTGGAAGACA	GTTGAGACCGCTGCCCGTTTAG
c51606	CCAATCGCAGTGCCAGTTCTTC	AGAGCATCCTGTGTTCCGTTGT
c81941	GATGCGGCACAAGGAGACCAA	TGAGTCGTCGTCAGCACTACCT
c47345	GTCTCAGCGAGCAACAGATGGT	GCGAGCAAAGAGCAAGCACAT

## Results and discussion

### Seed germination

The mature seed of *G. elata* contains a globular-shaped embryo covered by a thin layer of seed coat. After 4 weeks of inoculation (Fig. 1), the seed had been infected by fungal hyphae and germinated. The embryo enlarged further and resulted in the formation of mycorrhizal protocorms (early-satge, EP). Afterward, the protocorm elongated further and the shoot tip became visible (late-stage, LP).

### Proteome profiles

Unfortunately, it was technically impossible for us to remove the intracellular fungal hyphae (*Mycena*) from the protocorms. This meant that symbiotic cells contained transcripts produced by both partners (*G. elata* and *M. dendrobii*). Therefore, RNA from the *Mycena* library was used for establishing *Mycena* reference transcriptome. All of *M. dendrobii* reads derived from protocorm were removed by mapping all reads against the *Mycena* reference transcriptome. The clean reads from *G. elata* were *denovo* assembled into transcripts. We identified 139,756 unigenes. Among them, 9908 unigenes were able to translate into protein sequences and use for establishing the *G. elata* unigene translation database. The bioinformatics analyses were selected as reported in the previous studies (Liu et al. 2015a, b; Wang et al. 2016; Zeng et al. 2017). In our results, 3787 proteins were identified and quantified from our self-built database (unigene translation database) at a false discovery rate (FDR) of 1%. By analyzing, our self-built database was obviously suited for proteomic analyses.

**Fig. 2:**
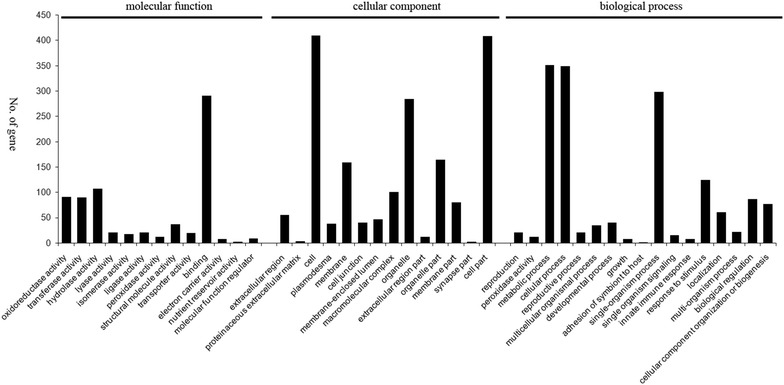
Go categorization of the total proteins

**Fig. 3:**
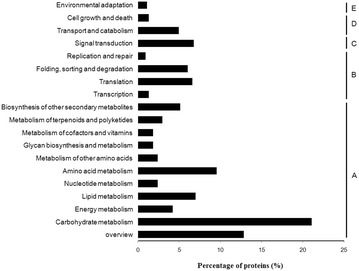
KEGG annotation of the total proteins. *A* Cellular processes; *B* Environmental information processing; *C* Genetic information processing; *D* Metabolism; *E* Organismal systems

Based on GO annotation (Fig. 2), the proteins were functionally classified according to the BP, CC, and MF categories and their subcategories. The largest subcategories for each functional group were as follows: “binding”, “hydrolase activity”, “transferase activity” and “oxidoreductase activity” for MF; “cell”, “cell part” and “organelle” for CC; and “cellular process”, “metabolic process”, “single-organism signaling” and “response to stimulus” for BP. According to KEGG (Fig. 3), most of the proteins were assigned to “signal transduction”, “translation”, “Amino acid metabolism” and “carbohydrate metabolism” pathways.

### Differentially accumulated proteins (DAPs)

**Fig. 4:**
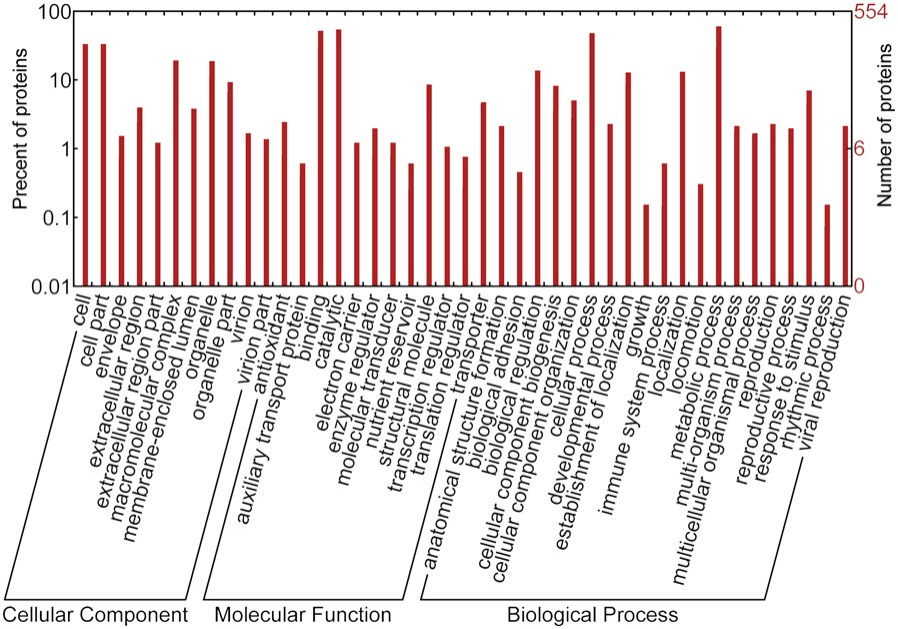
Go categorization of the differentially accumulated proteins

**Fig. 5:**
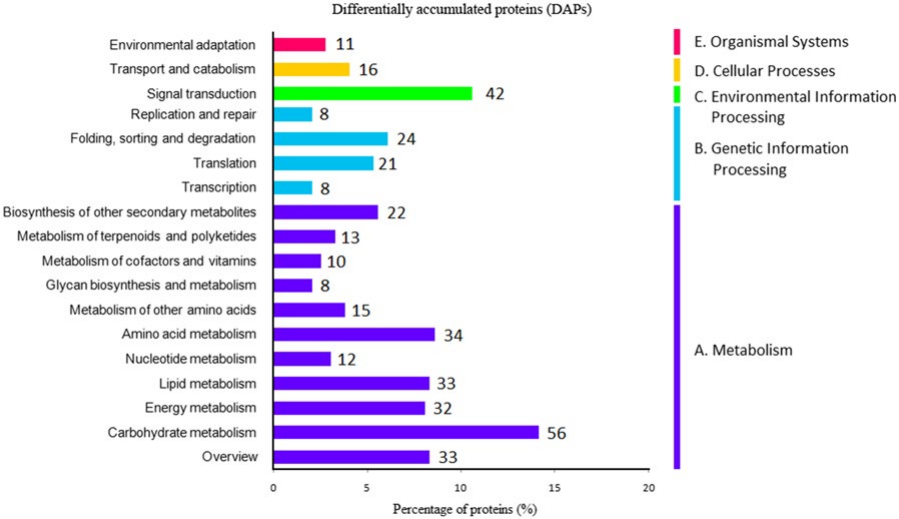
KEGG annotation of the differentially accumulated proteins

Compared to EP, 599 proteins were differentially accumulated in LP. Among these proteins, 321 proteins were significantly up-regulated, and 278 proteins were significantly down-regulated in protocorm. Based on GO analyses (Fig. 4), DAPs were functionally classified according to the BP, CC, and MF categories and their subcategories. The largest subcategories for each functional group were as follows: “cellular process”, “metabolic process”, and “response to stimulus” for BP; “cell”, “cell part” and “organelle” for CC; “binding”, “catalytic”, and “structural molecule” for MF. According to KEGG (Fig. 5), most of DAPs were assigned to “signal transduction”, “translation”, and “carbohydrate metabolism” pathways. Our results indicated that fungal colonization altered the metabolic processes of *G. elata* and could disrupt the balance between *Mycena* and *G. elata* during mycorrhizal symbiotic germination.

### Putative genes involved in plant defense

In general, the defense mechanism of the host plant is established after fungi infection, including the synthesis and accumulation of reactive oxygen species (ROS), phytoalexins and pathogenesis-related proteins. Recent studies have suggested that activation of defense mechanisms of rice plants by mycorrhizal fungi (*Glomus intraradices*) did not affect arbuscular mycorrhizal symbiosis (Campos-Soriano et al. 2010). Mycorrhizal fungi have evolved the capacity to circumvent defense mechanisms that are controlled by the plant’s immune system. However, our results indicated that defensive reaction could disrupt the balance the mycorrhizal symbiosis between *Mycena* and *G. elata* during germination. Here, we identified a number of proteins involved in plant defense (Table 2). Most of them were significantly up-regulated in the late-stage of protocorm. Therefore, *G. elata* defended itself against the colonization of *Mycena* in protocorm and interrupted the mycorrhizal symbiosis. In addition, *Mycena* fungi do not have the ability to suppress, neutralize, or evade the plant defense response.

**Table 2:** Defense genes involved in symbiotic germination of *G. elata*

Protein ID	Ratio change	P value	Uniprot ID	Reference gene
c54199	6.36	2.47E−02	Q9ZT66	Endoglucanase
c51606	3.76	2.52E−02	P00434	Peroxidase
c48836	3.34	3.16E−02	Q05736	Pathogenesis-related protein 1
c47606	2.96	3.81E−02	Q9FJZ9	Peroxidase
c81881	2.38	1.44E−05	Q9SW70	Stress-related protein
c81784	2.09	2.11E−05	O23814	Phospholipid hydroperoxide glutathione peroxidase
c41136	2.04	1.41E−04	Q6ZJJ1	l-Ascorbate peroxidase
c29958	1.77	2.10E−03	F4IXW2	Brefeldin A-inhibited guanine nucleotide-exchange protein
c47345	1.61	2.00E−03	Q10716	Cysteine proteinase
c81941	1.59	2.36E−03	Q7XQP4	Serine/threonine-protein kinase

Pathogenesis-related (PR) proteins are proteins produced in plants in the event of a pathogen attack. In our result (Table 2), a pathogenesis-related protein (PR1) was significantly up-regulated in LP compared with EP. In general, PR genes were strongly induced by fungal inoculation. For instance, after infection by fungi, PR1 proteins accumulate in maize seedlings that are primarily in contact with the pathogen and, as a second barrier, in papillae in the inner parts of the infected tissue. Several studies that have overexpressed PR genes have demonstrated that the role of PR proteins in plant-pathogen interactions was to enhance resistance to fungi (Maschietto et al. 2016). Moreover, PR proteins showed a broad-spectrum resistance to infection by bacterial and fungal pathogens. They displayed a basal expression level of endogenous defense genes and stronger and quicker defense responses during fungal infection (Ozgonen et al. 2009).

A recent molecular study by Perotto et al. (2014) indicated that none of the wound/stress-related genes were significantly up-regulated in mycorrhizal tissues (*Serapias vomeracea* infected with *Tulasnella calospora*). Meanwhile, Girlanda et al. (2011) demonstrated that *S. vomeracea* was a typical terrestrial orchid in the Mediterranean (partial mycoheterotrophic). In contrast, one stress-related protein was remarkably up-accumulated in the LP of *G. elata*. Our investigation from *G. elata* (fully mycoheterotrophic) suggested that fungal colonization triggered plant defense responses.

Previous studies have suggested that infection stress is accompanied by the production of ROS (H_2_O_2_, superoxide anion, etc.) in organisms (Liu et al. 2015a; Maschietto et al. 2016). The induction of enzymes, such as superoxide dismutase, peroxidases and catalases, could be involved in the protection of tissues against oxidative damage under infection conditions. The major functions of peroxidase include removal of H_2_O_2_, oxidation of toxic reductants, and response to stress, such as wounding, pathogen attack and oxidative stress. We identified that four peroxidases were up-accumulated in protocorms of *G. elata*. Among them, l-ascorbate peroxidase plays a key role in H_2_O_2_ removal (Teixeira et al. 2004). In our results, the LP of *G. elata* showed a constitutive higher level of L-ascorbate peroxidase, which was significantly increased after *Mycena* inoculation, contributing to efficient H_2_O_2_ scavenging. In addition, one up-regulated protein, phospholipid hydroperoxide glutathione peroxidase, protects cells and enzymes against oxidative damage, by catalyzing the reduction of H_2_O_2_, lipid peroxides, and organic hydroperoxide, by glutathione (Sugimoto et al. 1997).

Interestingly, one serine/threonine-protein kinase was found to be highly expressed in the LP tissues. MAPKKK serine/threonine-protein kinase confers sensitivity to various pathogens. This is required for resistance to some hemibiotrophic/necrotrophic fungal pathogens through the induction of defensin expression, probably by repressing MYC2, an inhibitor of defensin genes. Together with KEEP ON GOING protein, MAPKKK serine/threonine-protein kinase may regulate endocytic trafficking and/or the formation of signaling complexes on trans-Golgi network early endosome vesicles during stress responses (Gu and Innes 2011; Hiruma et al. 2011).

We also found two proteins up-accumulated in late-stage protocorms from subcategory “response to biotic stimulus” based on GO analyses. One protein (Brefeldin A-inhibited guanine nucleotide-exchange protein) plays a broad role in PAMP-triggered immunity, effector-triggered immunity, and salicylic acid-regulated immunity (Nomura et al. 2011). The other protein (cysteine protease) plays a role in immunity, senescence, and biotic and abiotic stress and may be involved in immunity against the necrotrophic fungal pathogen (Shindo et al. 2012).

The study of glucanase is hot topic in plant genetic engineering of disease resistance, and great progress on this subject has been made in the past few years (Day and Graham 2007). Glucans are major components of fungal cell wall. We analyzed hydrolases involved in the degradation of glucans. According to the results, one endoglucanase from *G. elata* protocorm was expressed to a great extent after *Mycena* infection. This protein could be involved in fungal cell wall hydrolysis.

### qPCR analysis of putative genes

**Fig. 6:**
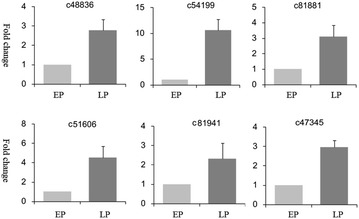
Quantitative PCR validations of six genes that were differentially expressed between the early-stage protocorms (EP) and the late-stage protocorms (LP)

We performed quantitative PCR analysis on 6 selected genes putatively involved in plant defense. The results of qPCR related to expression changes of these genes are shown in Fig. 6. In our results, most of the genes showed high gene expression in LP, and low gene expression in EP during the developmental process of seed germination of *G. elata*. In future, the expression of putative proteins in mycorrhizal symbiotic germination will be confirmed by the western blot.

## Conclusion

In conclusion, analysis of differentially accumulated proteins based on LC–MS/MS platform was a powerful method for investigating putative proteins involved in plant-fungus interactions. In the study, fungal colonization altered the metabolic processes of *G. elata*. We also analyzed pathogenesis-/stress-related proteins, peroxidases, and serine/threonine-protein kinase produced in the process of plant defense. These results indicated that the metabolic change and defense response of *G. elata* and could disrupt the balance between *Mycena* and *G. elata* during mycorrhizal symbiotic germination.
